# Pedals possible: a pilot study of adaptive cycling as a school-based rehabilitation intervention for students with disabilities

**DOI:** 10.3389/fped.2024.1463838

**Published:** 2025-01-15

**Authors:** Julia S. Brennan, Everette Keller, Elizabeth Humanitzki, Jessica Nichole Wade, Chad Catledge, Stephen Houston, Jonathan Beall, Cynthia B. Dodds

**Affiliations:** ^1^ Department of Health Science and Research, College of Health Professions, Medical University of South Carolina, Charleston, SC, United States; ^2^ Department of Public Health Sciences, College of Medicine, Medical University of South Carolina, Charleston, SC, United States; ^3^ Lancaster County School District, Lancaster, SC, United States; ^4^ Lancaster County Breakfast Rotary Club, Lancaster, SC, United States; ^5^ Heather’s Ride, Greenville, SC, United States; ^6^ Department of Rehabilitation Science, College of Health Professions, Medical University of South Carolina, Charleston, SC, United States

**Keywords:** adaptive cycling, school-based rehabilitation, goal attainment scaling, developmental delay, autism, physical activity, pediatric rehabilitation, community-based participatory research

## Abstract

**Introduction:**

Pediatric therapists in school-based practice can incorporate exercise promotion through adaptive cycling for children with disabilities who experience high levels of sedentary behavior and low levels of moderate to vigorous activity.

**Methods:**

The impacts of an adaptive cycling pilot program for children with disabilities were investigated through a community-based participatory study. During an eight-week intervention, students had a goal of riding adaptive cycles three times a week for twenty minutes. Using a pre-and post-test design, primary outcomes included individualized goal attainment scaling (GAS) linked to students' individualized education plans (IEP) and the 6-minute cycling test (6MCT) measuring cycling distance. Secondary outcomes included cycling duration over time, assistance levels for pedaling and steering, a “happiness scale”, and overall program satisfaction of parents and teachers. To prevent harm, pain behavior was examined using the Faces, Legs, Activity, Cry, Consolability (FLACC).

**Results:**

Cycling had a positive impact on students with disabilities. No increased levels of pain behavior or adverse events were reported. Individual GAS T-score means significantly improved to 0.24 and program effectiveness achieved a T-score value of 50.53. The mean distance of 6MCT increased from 728.95 feet to 880.5 feet. Secondary measures also documented significant improvement. Parents and teachers reported high overall satisfaction.

**Discussion:**

Adaptive cycling can incorporate needed physical activity into the school day and also support the achievement of IEP goals, physical activity capacity, and emotional happiness. Scaling adaptive cycling programs for children with disabilities should be considered an excellent opportunity for educational growth, health, and well-being.

## 1 Introduction

The recommended dosage of physical activity (PA) for school-aged children is 60 minutes a day as recommended by Healthy People 2030', which is a United States Department of Health and Human Services program that sets national objectives for improving health and well-being of its people (1, 2). The current proportion of children in the United States achieving this level of daily PA is low at 23.6% with an existing Healthy People 2030 objective seeking to increase the proportion of children meeting the 60 minute dosage (3). It is important to recognize that PA levels of children with disabilities trend even lower as they demonstrate high levels of sedentary behavior and low levels of moderate to vigorous PA. Thus, identifying realistic PA opportunities for this often under-represented population is essential (4). Since 1973, United States' federal regulations have supported the inclusion and participation of children with disabilities in public school programming, including physical education (5). Since children regularly attend school, improving PA opportunities within schools is reasonable.

Adaptive cycling is one such physical activity that has been incorporated into the school day (6), as well as clinical and home environments (7, 8). Existing evidence on adaptive cycling for children with disabilities is limited with most studies having been conducted in clinical environments rather than in natural childhood settings such as home or school. Preliminary evidence suggests that adaptive cycling may produce benefits including improved muscle strength, gross motor function, and achievement of individualized goals (7, 8). In the school setting, adaptive cycling has resulted in individualized goal achievement in a small sample of children with cerebral palsy (6). Strategies to initiate, sustain, and extend adaptive cycling opportunities in children with disabilities are needed although ideal training dosages have yet to be determined (8, 9).

To continue investigating the possible benefits of adaptive cycling programs in schools, this study used a community based participatory research (CBPR) model which allowed researchers to engage in an equal partnership with community entities. This ensured that the “interventions created responded to the community's needs” (10). The school-based environment provides an avenue for implementing CBPR studies in child and youth populations (11–17) with findings focusing on physical or social health, and some with a specific focus on physical activity (11, 12). Within existing evidence, one CBPR study focused on cycling, although not adaptive cycling, in a school environment (17).

This CBPR study aimed to investigate the impact of a school-based adaptive cycling program entitled “Pedals Possible” on children with disabilities within the Lancaster County School District located in South Carolina. It is hypothesized that participation in 20 minutes of physical activity over three days a week will improve educational goals, physical capacity, and emotional well-being in children with disabilities in a public school setting.

## 2 Methods

### 2.1 Human subject statement

This study involving human participant's was reviewed and approved by Lancaster County School District (LCSD). The Medical University of South Carolina (MUSC) had access only to de-identified data, so; Institutional Review Board approval was not necessary. (See Supplementary Material 1.5 for certification tool.)

### 2.2 Study design

In this CBPR model cohort study, a pre- and post-test design was implemented and measured the primary outcomes of individualized educational plan goals using goal attainment scaling methodology and cycling distance captured by the six-minute cycling test. A repeated measures design measured pain behaviors, happiness, assistance level and daily cycling duration (physical activity capacity). Program satisfaction, student social/school engagement, and physical activity survey data reported by parents and teachers were collected following completion of the 8-week intervention. Roles of CBPR partners and outcome measures are further described in upcoming sections of this paper.

#### 2.2.1 Community-based participatory partners

The four community partners who collaborated on this study included LCSD, Lancaster County Breakfast Rotary Club, Heather's Ride and MUSC College of Health Professions. The Lancaster County Breakfast Rotary Club established the Pedals Possible program which supported funding and classroom placement of the adaptive cycles and safety equipment. Heather's Ride (https://heathersride.org/), a non-profit organization based out of Greenville, South Carolina, selected and procured the adaptive cycling and safety equipment. Researchers from MUSC provided study funding, coordination, methodological support, data analyses and dissemination.

#### 2.2.2 Site and participants

Following recommendations of the school-based partner, inclusion criteria amongst the CBPR team were set as (1) any child within LCSD special education programming, (2) between the ages of 3 and 21 years, (3) and considered medically stable. Established exclusion criteria prevented a child's participation when they demonstrated (1) contraindications to increased physical activity (i.e., progressive neuromuscular disease), (2) elevated pain levels before preassessment, and (3) extreme behaviors that may cause harm. As agreed upon by the CBPR team, school administrators chose the special education classrooms from which to recruit children for study participation. LCSD elementary classrooms chosen to participate ranged from preschool to an equivalent of 5th grade. Parents and guardians of children within these classrooms were identified and approached by teachers or therapists for study participation. Twenty-one children with disabilities between the ages of three and ten years participated. See Table 1 for demographics.

**Table 1:** Demographics.

Demographics	Enrolled	Completed program
	Number of students (%/27)	Number of students (%/21)
Age		
3–4 years old	11 (40.7%)	9 (42.9%)
5–6 years old	8 (29.6%)	7 (33.3%)
7–8 years old	6 (22.2%)	4 (19%)
9–10 years old	1 (3.7%)	1 (4.8%)
Unknown	1 (3.7%)	0
Gender		
Male	22 (81.5%)	17 (80.9%)
Female	5 (18.5%)	4 (19.1%)
Race		
White/Caucasian	16 (59.3%)	12 (57.1%)
Black/African American	4 (14.8%)	4 (19.1%)
Hispanic/Latino	3 (11.1%)	3 (14.3%)
Asian	3 (11.1%)	2 (9.5%)
Other/Unknown	1 (3.7%)	0 (0%)
Ambulatory status		
Independent ambulator	26 (96.3%)	20 (95.2%)
Ambulates with assistive device	0	0
Wheelchair user	1 (3.7%)	1 (4.8%)
Diagnoses		
Developmental delay	9 (33.3%)	9 (42.9%)
Cerebral palsy	0	0 (0%)
Autism	13 (48.1%)	9 (42.9%)
Genetic disorder (Down Syndrome, Williams Syndrome, etc.)	4 (14.8%)	3 (14.3%)
Unknown	1 (3.7%)	0

#### 2.2.3 Cycling equipment

**Figure 1:**
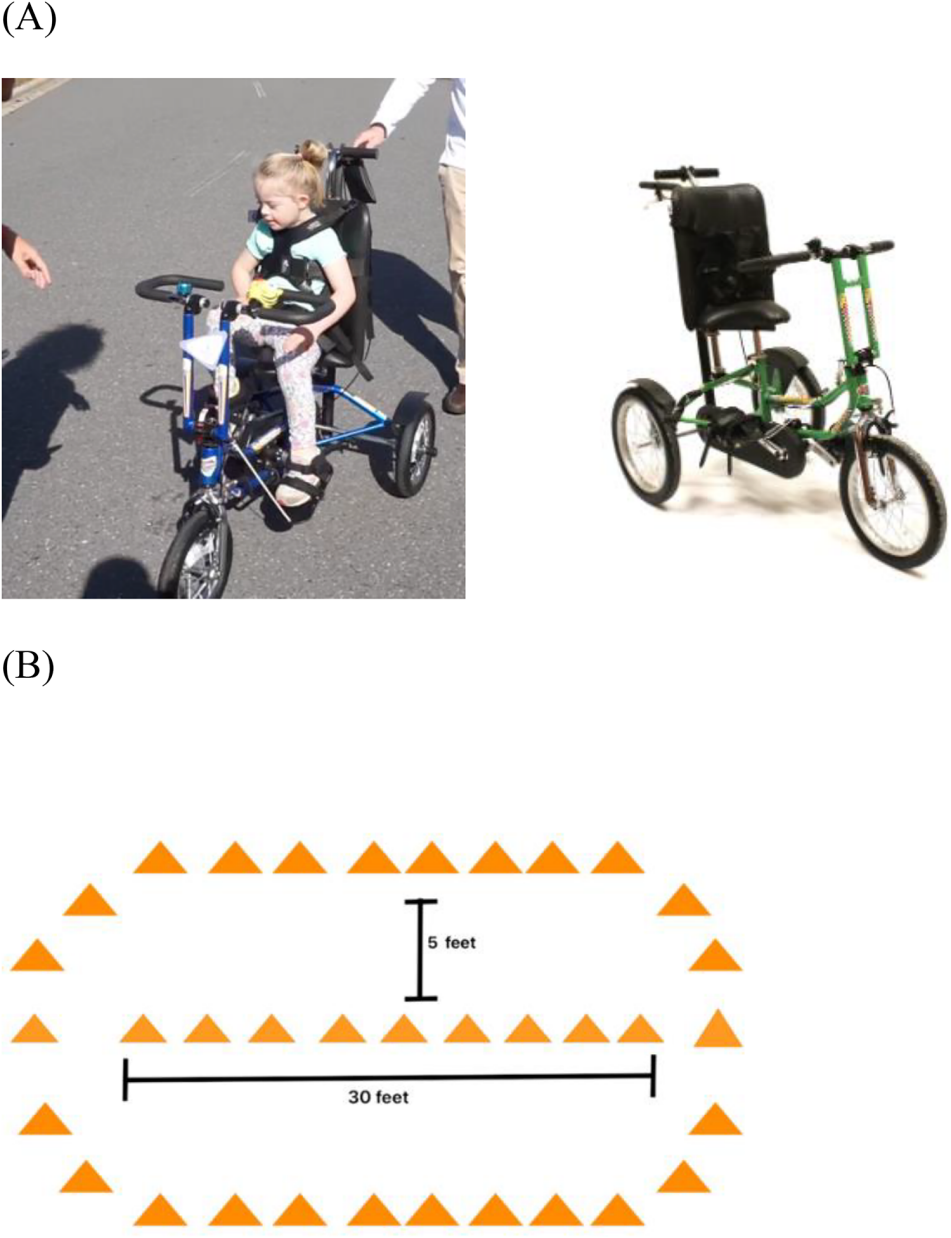
**(A)** Adaptive cycles (18). **(B)** Six minute cycling test course. Image of the cycle: Reprinted with permission from Freedom Concepts Inc, https://freedomconcepts.com/our-products/discovery-series/dcp-16/ (Accessed Jun 17, 2024), © Copyright Freedom Concept.

Discovery Series adaptive cycles from Freedom Concepts (i.e., DCP mini, DCP12, and DCP16) (https://freedomconcepts.com/our-products/discovery-series/dcp-12/) were used in this study. The features and sizes of the Discovery cycle met the heterogeneous needs of the participating children with disabilities. Standard features of the Discovery Series included: adjustable seat height, lockable hoop handlebars, low back seating system for postural support, large pedals/footplates with straps, direct drive gearing for improved propulsion power, and seatbelts (i.e., lap belt and chest belt) to promote safety. To address safety concerns, cycles were equipped with caregiver controls and helmets were available in a variety of sizes. An example of the cycle can be found in Figure 1A.

### 2.3 Intervention overview

Students participated in adaptive cycling for eight weeks with a goal of completing three, twenty- minute sessions each week for a total of twenty-four sessions. At times, school holidays and student absences interrupted the eight weeks of intervention. In these cases, up to two additional weeks were allotted to optimize the student's potential to complete twenty-four sessions. These sessions were completed during class, recess, or school-based therapy sessions. If a student was unable to cycle for twenty minutes, he/she was allowed to participate with the goal of attempting to reach the twenty minutes duration at some point across or by the end of the eight weeks. With training and guidance from the MUSC study coordinators, LCSD teachers, classroom assistants, or school-based physical or occupational therapists guided each cycling intervention. These individuals were familiar with each child that they assisted during the eight weeks of cycling. Classrooms were able to support one to two students' participation at a time and enrollment into the program occurred on a rolling basis across the school year.

The school-based physical therapists (PT) played an important and additional study role. After completing two on-site trainings with the MUSC study coordinators and researchers, they collected pre- and post-assessment data for the primary outcomes, served as communicators and liaisons between MUSC researchers, LCSD administrators, teachers and other school-based personnel, and maintained adaptive cycling equipment. Across the length of the study, LCSD school-based PTs met virtually twice a month with MUSC study coordinators and researchers to collaborate and/or problem solve any study matters and challenges related to procedures, data collection, and equipment.

### 2.4 Outcome measures

As recommended with CBPR, study tools needed to fit the characteristics of the community, which in this case was a heterogenic sample of students with disabilities enrolled in special education classrooms. Special education goals commonly address and measure gross motor, fine motor, selfcare, communication, behaviors, and social functioning domains. As such primary outcomes were reflective of these domains. School administrators, teachers, school-based PTs, stakeholders from Pedal Possible, Heather's Ride, and MUSC researchers reached consensus on chosen outcome measures, which helped ensure study compliance.

#### 2.4.1 Primary outcomes

The primary outcomes were collected before and after the eight-week cycling intervention.

Goal Attainment Scaling (GAS) was used to objectively measure individual student progress towards Individualized Educational Plan (IEP) goals. Effective use of GAS has been demonstrated in the school setting for children with disabilities (19, 20) and more specifically in a school-based adaptive cycling intervention for children with cerebral palsy (6). Based on the bell-shaped curve, GAS uses a five-point scale with raw scores ranging from −2 to +2 (−2, −1, 0, 1, 2) to predict expected goal outcomes and score progress. The “−2” value serves a baseline that represents current level of performance. The “0” value is defined as the expected level of performance. Goal Attainment Scaling methodology requires that only one variable within the goal is measured and increments between “−2” and “+2” must be equivalent. Examples of GAS are available in the Supplemental Material, as part of the Pre- and Post-intervention measures in Section 1.3. When used to measure change and score GASs, in this case at the end of eight-weeks, raw scores from one or more GASs are used to calculate T-scores. These T-scores can be reflective of individual or collective improvement including overall program effectiveness (21, 22). For this study, T-scores were calculated for each participants' one to three individual goals and the broader program effectiveness which included all study related GASs. Reliability of GAS is strongest when an interprofessional team is in place to establish GASs (23, 24). Thus, teachers, school-based PTs, and MUSC study coordinators and researchers worked collaboratively and consistently each week to establish, measure, and score each participant's one to three unique, individualized GASs.

The Six-minute cycling test (6MCT) was conceptualized from the six-minute walk test (6MWT) which measures physical capacity (25, 26). The 6MCT was standardized with a “cone course” (see Figure 1B) that was placed in a gym or lobby space of each school. Students were timed as they completed laps of the course and total distance in feet was measured using a Zozen collapsible measuring wheel. School PTs were trained by MUSC study coordinators and administered all 6MCT.

#### 2.4.2 Secondary outcome measures

Apart from the satisfaction survey, the secondary outcome measures were collected each session.

The Faces, Legs, Activity, Cry, Consolability (FLACC) behavior tool (27) is a validated pain behavior scale used for children who are non-verbal (28, 29). The primary reason for the inclusion of the FLACC was to mitigate harm by establishing a stopping and/or no-go criteria before and during the cycling intervention. Trained in the FLACC by school-based PTs, teachers, teaching assistants, or therapists who guided the cycling intervention for the day scored the FLACC before, during and after each session. Children did not participate or stopped participating in cycling if FLACC scores reached a value greater than eight.

Assistance level provided to each participant by teachers, teaching assistants or therapists during cycling session was collected for both pedaling and steering of the adaptive cycle. Levels of assistance were defined by the following: no assistance, minimum assistance of 25% or less support, moderate assistance of between 25% and 75% support, and maximum assistance of 75% or more support.

Session duration and cycling duration were collected. The overall session duration involved all tasks related to the cycling session such as transferring on and off the cycle, adjusting the cycle, allowing participants to become familiar with the cycle, donning/doffing safety equipment (i.e., helmet), and riding the cycle itself. The actual time spent cycling during session was defined as the cycling duration. School staff facilitating the cycling session documented the session and cycling durations. Session and cycling durations provided an indication of each participants' physical capacity.

A Happiness Scale was used to understand emotional changes that occurred across the adaptive cycling session. Before and after each cycling session, the Happiness Scale was visually presented to each participant, and he/she pointed to or circled the facial expression that most closely corresponded to their feelings. If the student was unable to self-report, the teacher, teaching assistant or therapist guiding the day's session, served as the proxy and scored the happiness scale. An example of the happiness scale is included in Supplementary Material 1.4 “Daily Flowsheet”.

A program specific, Likert scale surveys was created to understand program satisfaction, family or classroom social engagement, and changes in physical activity. With input from CBPR members, survey questions were created by study coordinators. Surveys were distributed to classroom teachers and parents of participating children following completion of the program. See Supplementary Materials 1.1–1.2 for the surveys.

### 2.5 Procedures

Lancaster County School District administration chose the special education classrooms that participated in the program. Administration from the district obtained parental informed consent and participant assent that allowed children to enroll in the study. Teachers and the three school-based PTs selected the students from each classroom to participate.

Primary outcome measures were collected by the school-based PTs within two weeks before the start and two weeks after the finish of the eight week intervention.

The secondary, repeated-measure outcomes were collected before and after each participant's cycling session. This data was recorded by a teacher, teaching assistant, or school-based therapists guiding the session.

Surveys were provided and collected from teachers and parents at the end of the study programming.

#### 2.5.1 Data collection

All primary and secondary outcomes were collected on paper using two study specific flowsheets, a pre- and post-intervention flowsheet and a pre- and post-session flowsheet. These were created by the MUSC coordinators and can be found in Supplementary Materials sections 1.3, 1.4. Data on the flowsheet was considered de-identified. The MUSC study coordinators provided each school-based PT with a hardcopy pre- and post-intervention flowsheet to collect primary outcomes. For secondary outcomes, each classroom was provided a binder with hardcopy pre- and post-session flowsheets. Throughout the length of the study, these de-identified flowsheets were scanned by the school-based PTs and securely sent to the research team by email. The research coordinator reviewed all flowsheets to ensure procedures were followed and to clarify discrepancies. Study coordinators transferred this data to a database on a MUSC secure server. At the end of each school semester, binders were returned and stored at MUSC.

### 2.6 Statistical analyses

Demographic and outcome data is summarized in Tables 1, 2. The primary research question was to assess how participation in the adaptive cycling pilot program impacts student's individualized goals (i.e., GASs) and the physical capacity (i.e., 6MCT). For each of these outcomes a paired *t*-test compared the pre- and post-test values, where the null hypothesis stated that the mean difference in the outcome from the pre- to post-visit was 0. Each secondary outcome measure (e.g., happiness scale, assistance level, FLACC, and surveys) was modeled using a multivariable mixed model regression framework to assess whether the time spent in the cycling intervention demonstrated improvement from the initial visit across the eight weeks of study participation. For binary outcomes, a binomial distribution with a logit link was assumed. For ordinal variables, proportional odds model was estimated. For, continuous outcomes, a multivariable linear mixed model was implemented, and for ordinal outcomes, a two-way repeated measures ordinal regression model was used.

**Table 2:** Secondary outcomes.

	1st session	Last session
Pedal assistance levels *n* (%)		
No assistance	6 (29%)	13 (62%)
Minimum assistance	4 (20%)	2 (10%)
Moderate assistance	4 (20%)	4 (19%)
Maximum assistance	7 (33%)	2 (10%)
Steering assistance levels *n* (%)		
No assistance	0 (0%)	3 (14%)
Minimum assistance	1 (5%)	3 (14%)
Moderate assistance	7 (35%)	5 (24%)
Maximum assistance	13 (62%)	10 (48%)
Cycling duration mean (SD)		
In minutes	18.31 (4.63)	21.25 (7.57)
Overall impression of program: *n* (%)^a^		
Strongly disagree		0 (0%)
Disagree		0 (0%)
Neutral		1 (3.2%)
Agree		11 (35.5%)
Strongly agree		19 (61.3%)
Overall impression of school engagement *n* (%)^b^		
Strongly disagree		0 (0%)
Disagree		0 (0%)
Neutral		3 (15.8%)
Agree		13 (68.4%)
Strongly agree		3 (15.8%)
Overall impression of family/community engagement *n* (%)^c^		
Strongly disagree		0 (0%)
Disagree		1 (8.3%)
Neutral		0 (0%)
Agree		10 (83.3%)
Strongly agree		1 (8.3%)
Overall impression of physical activity tolerance *n* (%)^a^		
Strongly disagree		0 (0%)
Disagree		0 (0%)
Neutral		4 (12.9%)
Agree		19 (61.3%)
Strongly agree		8 (25.8%)

*n*, number of students;%, percentage of students.

^a^ 31 parent/caregiver and teacher/therapist responses.

^b^ 19 teacher/therapist responses.

^c^ 12 parent/caregiver responses.

For each of the regression models, normality assumptions were assessed and confirmed by visualizing plots of residuals. *P*-values <0.05 were considered statistically significant. All statistical analyses were performed using R version 4.1.3. (v4.1.2; R Core Team 2021).

## 3 Results

### 3.1 Baseline demographic and clinical characteristics

A summary of participant's recruitment through enrollment is depicted in Supplementary Material 2.1. Reasons for drop out included: three due to classroom staffing shortage, one due to a social circumstance in the home, one due to absences, and one due to withdrawal from school. The baseline demographic and clinical characteristics of the remaining 21 participants in the study cohort are summarized in Table 1. Of the 21 participants, nine (42.9%) were diagnosed with developmental delay, nine (42.9%) were diagnosed with autism, and three (14.3%) were diagnosed with a genetic disorder. The goal was for each student participant to reach 24 intervention sessions over eight weeks with a 2 week extension if needed, the overall session attendance rate was 72.6% of those 24 sessions.

### 3.2 Primary outcomes

#### 3.2.1 Goal attainment scalings and six-minute cycling test

Goal attainment scalings were categorized across four educational-related categories: attention at 21%, communication at 15%, social function at 15%, and gross motor/physical activity at 49%.

**Figure 2:**
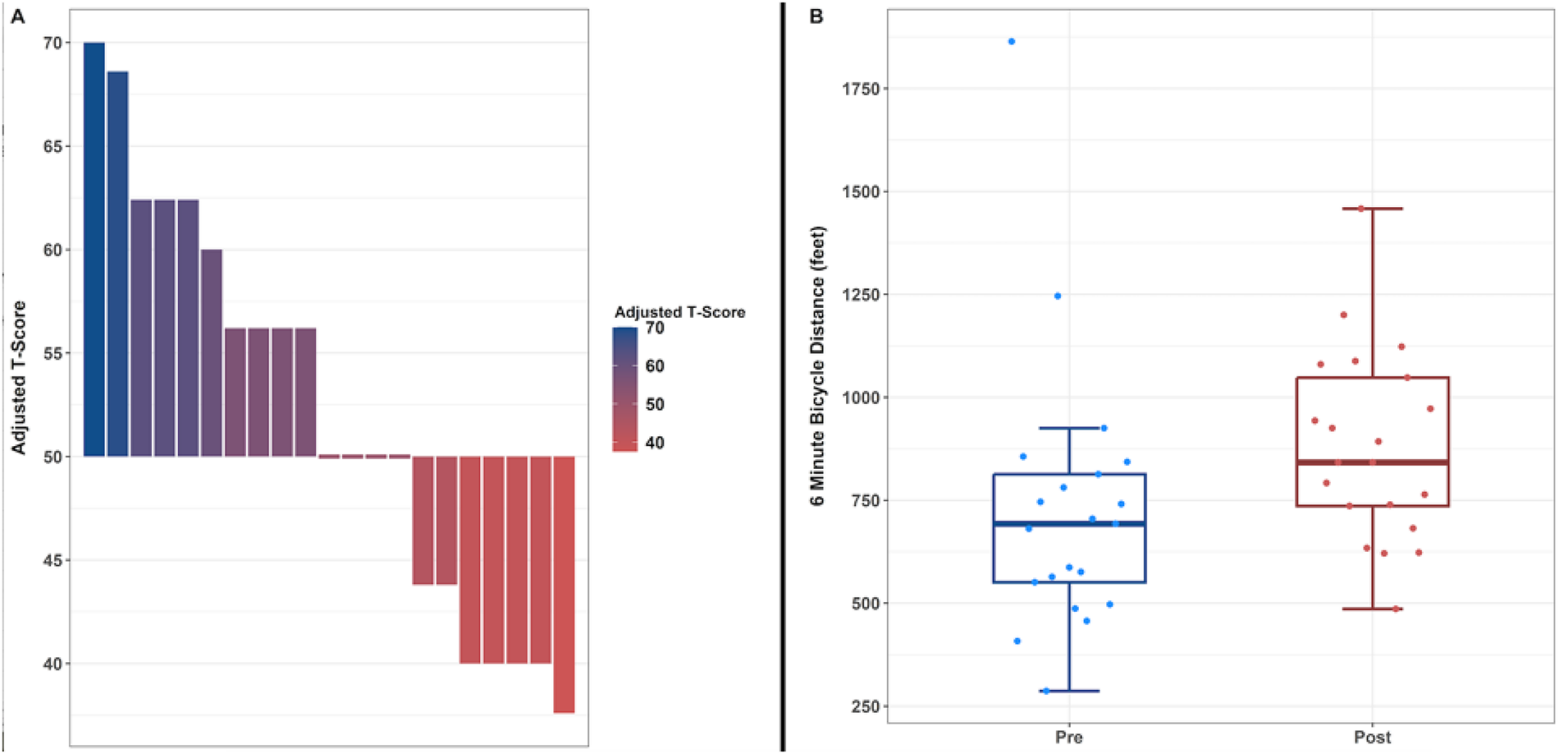
**(A)** Goal attainment scaling T-scores at post-assessment. **(B)** 6-minutes cycling test distances at pre and post testing.

The pre-test GAS raw score mean value was −2 with a T-score value of 27.25. The mean raw score post-test achieved 0.24 with corresponding T-score of 52.18. Examination of the paired pre- and post-GAS scores using a paired *t*-test yielded statistically significant difference (*p* < 0.05). Given initial GAS was at the minimum possible value of −2 for each participant, the statistically significant difference indicated that participation in the adaptive cycling program was associated with improved progress on students' IEP goals. This is depicted in Figure 2. The program effectiveness score which is reflective of all student's goal achievement was 50.53.

The 6MCT pre-test mean was 728.95 feet with a post-test value of 880.5 feet, resulting in a mean difference of 151.55 feet. (See Figure 2). The paired *t*-test indicated there was not sufficient evidence to suggest rejecting the null hypothesis that a difference between the pre- and post-visits existed (*p* = 0.051). However, given the clinically meaningful increase in the distance cycled as well as the near statistically significant *p*-value, we find that the utilization of the adaptive cycling program obtained one of the desired results of increasing physical activity levels in participing children with disabilities. (See Figure 2).

### 3.3 Secondary outcomes

Mean and standard deviation from secondary measures can be found in Table 2.

#### 3.3.1 Face legs activity cry consolability scale

The FLACC was used across the 8-weeks of cycling sessions to mitigate harm. Only one participant was not able to participate in a cycling session because of a FLACC score over the established threshold of 8. Within the model, FLACC scores obtained at the end of each individual session were estimated to be approximately 0.886 points lower than scores obtained prior to an individual session. This provides an indication that adaptive cycling did not increase pain and demonstrates that no harm occurred.

#### 3.3.2 Assistance level scale

The two-way repeated measures ordinal regression model examining the assistance level scale (pedaling and steering ability) as a function of repeated visits and found a statistically significant difference in pedal assistance across the 8-week duration of the study (*p* < 0.05). The degree of pedal assistance required for participants significantly decreased across the duration of intervention. There was not a statistically significance between visits and steering assistance.

#### 3.3.3 Happiness scale

A linear mixed regression model examined the happiness score as a function of when the score was measured at each individual session (at the start or at the end) and the interaction between when the score was measured at each individual session and visits. Happiness scores obtained after each individual session relative to being obtained at the start of each individual session were statistically significant (*p* < 0.05). Within the model, happiness scores obtained at the end of each individual session were estimated to be approximately 0.7 points higher than scores obtained prior to an individual session.

#### 3.3.4 Cycling duration

Cycling duration was recorded at each session to provide an indication on change in physical capacity of children with disabilities. Sessions varied in length of time; however sustained cycling was documented on average for 83.6% of a given session. A linear mixed-effects model accounting for repeated measures was used to examine whether there was an association between students' session and their cycling duration. Assuming a random subject effect of 0, cycling duration is expected to increase by approximately 0.0092 minutes (5.5 s) for each session a child attends. For a child that attends all twenty-four sessions, cycling duration was expected to increase by approximately 2.2 minutes (132 s) from prior to their 1st session to their 24th session. With a *p*-value of 0.012, this was a statistically significant result (*p* < 0.05).

#### 3.3.5 Surveys

Overall impressions (e.g., satisfaction, student social/school engagement, and physical activity) of the adaptive cycling program for children with disabilities, as assessed by teacher and parental surveys range from 0 (strongly disagree) to 5 (strongly agree). With regards to the overall impression of the adaptive cycling program, 96.8% of parents/caregivers and teachers indicated a positive agreement with 61.2% having a strong positive agreement. Additionally, 87.1% of parents/caregivers and teachers provided positive agreement that the participants' physical activity capacity improved, 84.2% of teachers had a positive agreement that the program improved school engagement, and 91.7% of parents/caregivers indicated a positive agreement that the program had a positive impact on the family and community engagement. In addition to positive findings from the surveys, qualitative statements further substantiated findings. One teacher commented that “Overall, (the child) is making huge gains in social emotional skills, including playing and communicating with peers, following directions, and using playground equipment”. Another noted emotional change, “He was always more calm and focused after his rides”. “Every day we see a wonderful improvement in (our child), his self-confidence has grown and he is starting to initiate play!” reported one parent. Another noted, “(our child) has grown active in her playing and in her verbal ability”.

## 4 Discussion

This school-based cycling study demonstrated positive findings for children with disabilities. Not only did goal attainment scalings, aligned with individualized educational goals, display improvements, but physical capacity as measured by the 6MCT, session cycling durations, and levels of pedaling assistance did as well. This provides indications that school-based cycling programs are valuable, which is similar to findings from another school-based cycling program (6) and other general adaptive cycling programs for children with disabilities (7–9). Adaptive cycling is a realistic activity that can occur within a school-based environment in order to support PA in children with disabilities (4), contribute to the recommended PA dosage of 60 minutes a day (1), and improve the likelihood that a greater proportion of children in the United States reach recommended 60-minutes daily dosages (3).

Study findings documented improvements despite many participants not completing 24 sessions. Participants completed 72.6% of the 24 sessions, or a rate of 18 sessions total. While this may indicate that positive gains can be made with fewer cycling sessions, it would be a premature assumption. To identify a cycling dose response, minutes of cycling, assistance level considerations, fidelity of treatment, and environmental barriers and facilitators would need to be better documented and/or better controlled than this preliminary study was capable. Ideally, future studies will begin to examine some of these features. In the meantime, what can be stated is that a program targeting 20 minutes of adaptive cycling for 24 sessions for ambulatory children with disabilities improves individualized school-related goals, happiness, and physical capacity.

Students with disabilities demonstrated improved happiness as a result of participating in this school-based adaptive cycling program. During a time of heightened of mental health awareness for children with disabilities, especially in the wake of COVID-19 restrictions (30), this is a key finding. Recent evidence shows that mental health conditions are more common in young people with intellectual or developmental disabilites in comparison to the general population (31–33). These findings support that one potential action to contribute to improvements in child mental well-being is to increase PA and facilitate its positive effect on behavior and emotional problems (34).

Not only did this school-based PA intervention not cause harm, it was also feasibly carried out in a school setting, which supports recommendations from the Centers for Disease Controls “Heathy Schools” initiative. Five components of the this comprehensive school physical activity program include: physical activity before and after school, physical education, physical activity during school, family and community engagement, and staff involvement (35). Expansion of this adaptive cycling program offers multiple avenues for growth across these five areas, such as incorporating cycling into a student with disabilities' commute between home and school, mobility within the school as an alternative to a wheelchair or stroller mobility, or as part of extracurricular and/or physical education programming.

Pediatric PTs working within school-based practice have capacity to change the trajectory of PA opportunities for children with disabilities! First, the greatest percentage of pediatric PTs work in the school-based practice (36), which if banded together, their impact on PA for children with disabilities could be remarkable. Secondly, pediatric PTs have expertise regarding children with disabilities and health promotion which better ensures that PA can be provided to the children properly and safely. Lastly, the school environment within which they work is a true participatory environment for children with disabilities. Collectively, these factors provide great capacity to generate systems change in implementing and achieving PA recommendations for children with disabilities.

As with many successful rehabilitation strategies for children with disabilities (37–39), an interdisciplinary (ID) approach is beneficial to creating success. This is true for school-based environment as well. In this study, the alliance between members of the LCSD school environment (i.e., teachers, teaching assistants, physical and occupational therapists, administrators) was key to successful programmatic implementation and results. These school-based physical therapists also mentioned that teamwork and support between disciplines empowered teachers and staff members which resulted in increased adherence to the proposed schedule of three sessions per week. Additionally, involvement from multiple disciplines also allows for dynamic and responsive problem solving as well as service delivery. This ID approach is common and expected within school-based practice; thus, implementation of similar programs across public school entities is a realistic possibility and would benefit children with disabilities.

A facilitator of success for this school-based program included utilization of a CBPR approach. All stakeholders played an essential role in creating a community which enriched the learning, health and well-being of children with disabilities as well as added to the evidence. In public school settings where financial resources are often limited, a CBPR approach may offer realistic opportunities to enhance achievement and health promotion of students with disabilities (11). In addition, as demonstrated in this and earlier studies (11–17), research support from academic entities and community partners can often provide the momentum to carry an initiative through in its entireity, including in the school setting. This project's CBPR relationship is ongoing and continuing with study dissemination, extramural grant submission and expansion into additional classrooms, school districts, and community recreational centers.

### 4.1 Limitations guiding future directions

The lack of a control group limits generalization of these findings; however, it may be challenging to implement control groups within the context of a public school which is designed to offer a free and appropriate education to all students. Designs of future studies may need to consider single subject design or crossover approaches that promote control group-like comparisons.

Although the 6MCT was founded on the well-validated 6-Minute Walk Test (25, 26) and was standardized in its administration, it is unvalidated which created a study limitation. It was used as an attempt to have a common measure of physical capacity in students of all mobility levels. Validation of the 6MCT in future studies would be constructive.

The aim of this school-based study was to examine the benefits of a regular physical activity program for children with disabilities. This study saw positive outcomes following participation in an adaptive cycling program in children with disabilities, but the study sample consisted mostly of ambulatory children with the majority having autism spectrum disorders. This is similar to what is currently found in the literature. For example, a scoping review of leisure time PA in children and adults with cerebral palsy, 49 studies were included, but only 17 involved individuals who used wheelchairs (40). Additionally, in two systematic reviews of PA in children with CP, all outcomes examined standing and walking (41, 42). The absences of non-ambulatory children with disabilities in this study may be reflective of the CBPR model and the important role that school district administrators had in selecting classrooms and children that participated. Knowing that physical activity levels are reduced for all children with disabilities and improving these levels may provide learning, health, and well-being benefits, it is essential that research efforts and PA opportunities are expanded and carried out. This includes the inclusion of nonambulatory children with disabilities.

## 5 Conclusion

Overall, this program demonstrates that adaptive cycling was a feasible intervention for those who participated in this study in the school environment, yielded benefits and caused no harm. Implementation of this program in the school environment offers children with disabilities an opportunity to participate in PA that is fun, meaningful and can easily be incorporated into their daily routine. Not only did the school-based setting provide an optimal site for conducting community based participatory research; the CBPR approach generated additional resources for the schools while producing valuble research findings and community engagement. Future projects would benefit from utilization of the school based setting to research PA for children with disabilities.

## Data Availability

The original contributions presented in the study are included in the article/Supplementary Material, further inquiries can be directed to the corresponding author.

## References

[1] U.S. Department of Health and Human Services. Physical Activity Guidelines for Americans. 2nd ed. Washington, DC: U.S. Department of Health and Human Services (2018).

[2] Garber CE Blissmer B Deschenes MR Franklin BA Lamonte MJ Lee I Quantity and quality of exercise for developing and maintaining cardiorespiratory, musculoskeletal, and neuromotor fitness in apparently healthy adults. Med Sci Sports Exerc. (2011) 43(7):1334–59. 10.1249/MSS.0b013e318213fefb 21694556

[3] US Department of Health and Human Services. Increase the proportion of children who do enough aerobic physical activity – PA-09 data. Increase the proportion of children who do enough aerobic physical activity - Data - Healthy People 2030. Available online at: https://health.gov/healthypeople/objectives-and-data/browse-objectives/physical-activity/increase-proportion-children-who-do-enough-aerobic-physical-activitypa09/data (accessed June 12, 2024)

[4] Verschuren O Peterson MD Balemans ACJ Hurvitz EA. Exercise and physical activity recommendations for people with cerebral palsy. Dev Med Child Neurol. (2016) 58(8):798–808. 10.1111/dmcn.13053 26853808 PMC4942358

[5] United States Department of Health, Education, and Welfare. Rehabilitation Act of 1973 (1973). s. 504.

[6] Daly C Moore CL Johannes S Middleton J Kenyon LK. Pilot evaluation of a school-based programme focused on activity, fitness, and function among children with cerebral palsy at GMFCS Level IV: single-subject research design. Physiother Can. (2020) 72(2):195–204. 10.3138/ptc-2018-0053 32494103 PMC7238939

[7] Thevarajah A Wallen M Imms C Lonsdale C Carey JJ Froude EH. Impact of adapted bicycle riding on outcomes for children and adolescents with disabilities: a systematic review. Dev Med Child Neurol. (2023) 65(4):456–68. 10.1111/dmcn.15446 36335550

[8] Armstrong EL Spencer S Kentish MJ Horan SA Carty CP Boyd RN. Efficacy of cycling interventions to improve function in children and adolescents with cerebral palsy: a systematic review and meta-analysis. Clin Rehabil. (2019) 33(7):1113–29. 10.1177/0269215519837582 30935240

[9] Carey JJ Toovey R Spittle AJ Imms C Shields N. Exploring adaptive cycling interventions for young people with disability: an online survey of providers in Australia. J Clin Med. (2023) 12(17):5523. 10.3390/jcm12175523 37685591 PMC10488225

[10] Community-based Participatory Research Program (CBPR). U.S. Department of Health and Human Services; (2024). Available online at: https://www.nimhd.nih.gov/programs/extramural/community-based-participatory.html (Accessed Jun 12, 2024).

[11] Zahnd WE Smith T Ryherd SJ Cleer M Rogers V Steward DE. Implementing a nutrition and physical activity curriculum in head start through an academic-community partnership. J Sch Health. (2017) 87(6):465–73. 10.1111/josh.12515 28463443

[12] Wright K Giger JN Norris K Suro Z. Impact of a nurse-directed, coordinated school health program to enhance physical activity behaviors and reduce body mass index among minority children: a parallel-group, randomized control trial. Int J Nurs Stud. (2013) 50(6):727–37. 10.1016/j.ijnurstu.2012.09.004 23021318 PMC3654538

[13] Wright K Suro Z. Using community-academic partnerships and a comprehensive school-based program to decrease health disparities in activity in school-aged children. J Prev Interv Community. (2014) 42(2):125–39. 10.1080/10852352.2014.881185 24702663

[14] Jacquez F Vaughn LM Wagner E. Youth as partners, participants or passive recipients: a review of children and adolescents in community-based participatory research (CBPR). Am J Community Psychol. (2013) 51(1–2):176–89. 10.1007/s10464-012-9533-7 22718087

[15] Laurence B Shara N Gonzalez F Harris D Edmonds T Grant-Mills D An approach to engaging schools in oral health initiatives: the Howard Meharry adolescent caries study (HMACS). J Health Care Poor Underserved. (2020) 31(1):35–42. 10.1353/hpu.2020.0006 32037315

[16] Eghbalnia C Sharkey K Garland-Porter D Alam M Crumpton M Jones C A community-based participatory research partnership to reduce vehicle idling near public schools. J Environ Health. (2013) 75(9):14–9. 23734527

[17] Davison K Bowling A Garcia J Wood B Hermesch R Prince J A cybercycling intervention to improve behavioral regulation and classroom functioning among children with behavioral health disorders: pragmatic randomized trial design for Manville moves. Contemp Clin Trials. (2016) 49:40–6. 10.1016/j.cct.2016.05.008 27261171

[18] Freedom Concepts DCP 16. (2023). Available online at: https://freedomconcepts.com/our-products/discovery-series/dcp-16/ (Accessed Jun 17, 2024).

[19] Kascak K Keller E Dodds C. Use of goal attainment scaling to measure educational and rehabilitation improvements in children with multiple disabilities. Behav Sci. (2023) 13(8):625. 10.3390/bs13080625 37622765 PMC10451652

[20] Effgen SK McCoy SW Chiarello LA Jeffries LM Bush H. Physical therapy-related child outcomes in school: an example of practice-based evidence methodology. Pediatr Phys Ther. (2016) 28(1):47–56. 10.1097/PEP.0000000000000197 27088686

[21] Kiresuk TJ Sherman RE. Goal attainment scaling: a general method for evaluating comprehensive community mental health programs. Community Ment Health J. (1968) 4(6):443–53. 10.1007/BF01530764 24185570

[22] Gaffney E Gaffney K Bartleson L Dodds C. Goal attainment scaling made easy with an app: GOALed. Pediatr Phys Ther. (2019) 31(2):225–30. 10.1097/PEP.0000000000000602 30907841

[23] Steenbeek D Ketelaar M Lindeman E Galama K Gorter JW. Interrater reliability of goal attainment scaling in rehabilitation of children with cerebral palsy. Arch Phys Med Rehabil. (2010) 91(3):429–35. 10.1016/j.apmr.2009.10.013 20298835

[24] Krasny-Pacini A Pauly F Hiebel J Godon S Isner-Horobeti ME Chevignard M. Feasibility of a shorter goal attainment scaling method for a pediatric spasticity clinic—the 3-milestones GAS. Ann Phys Rehabil Med. (2017) 60(4):249–57. 10.1016/j.rehab.2017.01.005 28365157

[25] Matos Casano HA Anjum F. Six-minute walk test. In: StatPearls [Internet]. Treasure Island (FL): StatPearls Publishing (2024). https://www.ncbi.nlm.nih.gov/books/NBK576420/35015445

[26] Li AM Yin J Yu CCW Tsang T So HK Wong E The six-minute walk test in healthy children: reliability and validity. Eur Respir J. (2005) 25(6):1057–60. 10.1183/09031936.05.00134904 15929962

[27] Merkel SI Voepel-Lewis T Shayevitz JR Malviya S. The FLACC: a behavioral scale for scoring postoperative pain in young children. Pediatr Nurs. (1997) 23(3):293–7. 9220806

[28] Malviya S Voepel-Lewis T Burke C Merkel S Tait AR. The revised FLACC observational pain tool: improved reliability and validity for pain assessment in children with cognitive impairment. Pediatr Anesth. (2006) 16(3):258–65. 10.1111/j.1460-9592.2005.01773.x 16490089

[29] Voepel-Lewis T Zanotti J Dammeyer JA Merkel S. Reliability and validity of the face, legs, activity, cry, consolability behavioral tool in assessing acute pain in critically ill patients. Am J Crit Care. (2010) 19(1):55–61; quiz 62. 10.4037/ajcc2010624 20045849

[30] Theis N Campbell N De Leeuw J Owen M Schenke KC. The effects of COVID-19 restrictions on physical activity and mental health of children and young adults with physical and/or intellectual disabilities. Disabil Health J. (2021) 14(3):101064. 10.1016/j.dhjo.2021.101064 33549499 PMC7825978

[31] Rydzewska E Hughes-McCormack LA Gillberg C Henderson A MacIntyre C Rintoul J Prevalence of sensory impairments, physical and intellectual disabilities, and mental health in children and young people with self/proxy-reported autism: observational study of a whole country population. Autism. (2019) 23(5):1201–9. 10.1177/1362361318791279 30328695

[32] Einfeld SL Ellis LA Emerson E. Comorbidity of intellectual disability and mental disorder in children and adolescents: a systematic review. J Intellect Dev Disabil. (2011) 36(2):137–43. 10.1080/13668250.2011.572548 21609299

[33] Emerson E Einfeld S. Emotional and behavioural difficulties in young children with and without developmental delay: a bi-national perspective. J Child Psychol Psychiatry. (2010) 51(5):583–93. 10.1111/j.1469-7610.2009.02179.x 20015191

[34] Borland RL Cameron LA Tonge BJ Gray KM. Effects of physical activity on behaviour and emotional problems, mental health and psychosocial well-being in children and adolescents with intellectual disability: a systematic review. J Appl Res Intellect Disabil. (2022) 35(2):399–420. 10.1111/jar.12961 34796601

[35] Physical Education and Physical Activity. Centers for Disease Control and Prevention; (2023). Available online at: https://www.cdc.gov/healthyschools/physicalactivity/index.htm (Accessed Jun 08, 2024).

[36] Effgen SK Kaminker MK. Nationwide survey of school-based physical therapy practice. Pediatr Phys Ther. (2014) 26(4):394–403. 10.1097/PEP.0000000000000075 25251792

[37] Steenbeek D Ketelaar M Galama K Gorter JW. Goal attainment scaling in paediatric rehabilitation: a report on the clinical training of an interdisciplinary team. Child Care Health Dev. (2008) 34(4):521–9. 10.1111/j.1365-2214.2008.00841.x 19154553

[38] Anaby DR Campbell WN Missiuna C Shaw SR Bennett S Khan S Recommended practices to organize and deliver school-based services for children with disabilities: a scoping review. Child Care Health Dev. (2019) 45(1):15–27. 10.1111/cch.12621 30264437

[39] Hall JB Chole D Pruitt TC Linkeman K. Caregiver perceptions of an interdisciplinary intensive therapy program: a qualitative study. Pediatr Phys Ther. (2023) 35(2):228–35. 10.1097/PEP.0000000000000994 36637445

[40] Lai B Lee E Kim Y Matthews C Swanson-Kimani E Davis D Leisure-time physical activity interventions for children and adults with cerebral palsy: a scoping review. Dev Med Child Neurol. (2021) 63(2):162–71. 10.1111/dmcn.14751 33241561

[41] O'Brien TD Noyes J Spencer LH Kubis HP Hastings RP Whitaker R. Systematic review of physical activity and exercise interventions to improve health, fitness and well-being of children and young people who use wheelchairs. BMJ Open Sport Exerc Med. (2016) 2(1):e000109. 10.1136/bmjsem-2016-000109 PMC5125427 27900176

[42] Reedman SE Boyd RN Elliott C Sakzewski L. ParticiPAte CP: a protocol of a randomised waitlist controlled trial of a motivational and behaviour change therapy intervention to increase physical activity through meaningful participation in children with cerebral palsy. BMJ Open. (2017) 7(8):e015918. 10.1136/bmjopen-2017-015918 28790038 PMC5629713

