# Supplementary material for: Pedals possible: a pilot study of adaptive cycling as a school-based rehabilitation intervention for students with disabilities
Source: Front Pediatr. 2025 Jan 15;12:1463838. doi: 10.3389/fped.2024.1463838 (PMC11776867; doi:10.3389/fped.2024.1463838)
Supplement: Supplementary file 1 [file Datasheet1.docx]

Supplementary Material

Pedals Possible: A Pilot Study of Adaptive Cycling as a School-Based Rehabilitation Intervention for Students with Disabilities

Julia S. Brennan^1*^, Everette Keller^2^, Elizabeth Humanitzki^1^, Jessica Nicole Wade^3^, Chad Catledge^4^, Stephen Houston^5^, Jonathan Beall^2^, Cynthia B. Dodds^6^

^1^Department of Health Science and Research, College of Health Professions, Medical University of South Carolina, Charleston, SC, United States, ^2^Department of Public Health Sciences, College of Medicine Medical University of South Carolina, Charleston, SC, United States, ^3^Lancaster County School District, Lancaster, SC, United States, ^4^Lancaster County Breakfast Rotary Club, Lancaster, SC, United States, ^5^Heather’s Ride, Greenville, SC, United States, ^6^Department of Rehabilitation Science, College of Health Professions, Medical University of South Carolina

*** Correspondence:**Julia S. Brennan
[schroeder@musc.edu](mailto:schroeder@musc.edu)

# Supplementary Data

## Teacher and Therapist Likert Scale

***Pedals Possible Teacher/Therapist Questionnaire***

**For the following phrases, please circle the response that you most identify with:**

**Overall Impression of Program**

1. The student had a positive experience with the school-based adaptive cycling program

Strongly Agree Agree Neutral Disagree Strongly Disagree

1. I would sign this student up for a program like this again.

Strongly Agree Agree Neutral Disagree Strongly Disagree

1. Based on my experience with this student, I would recommend this program to other school districts.

Strongly Agree Agree Neutral Disagree Strongly Disagree

**School Engagement**

1. The student has improved engagement in school-related daily tasks (self-care, educational tasks, classroom care, mealtimes, etc.)

Strongly Agree Agree Neutral Disagree Strongly Disagree

1. The student has improved participation with peers in a social capacity in the school environment

Strongly Agree Agree Neutral Disagree Strongly Disagree

1. This program was feasible for this student in this specific classroom.

Strongly Agree Agree Neutral Disagree Strongly Disagree

**Physical Activity**

1. The student’s ability to perform gross motor tasks has improved (walking, running, wheeling wheelchair, riding bike, etc.)

Strongly Agree Agree Neutral Disagree Strongly Disagree

1. This student has improved participation in physical activity during the day (sports, playing on playground, outside play, etc.)

Strongly Agree Agree Neutral Disagree Strongly Disagree

1. The student has an improved mood.

Strongly Agree Agree Neutral Disagree Strongly Disagree

Overall Comments: ____________________________________________________________________________________________________________________________________________________________________________

## Parent/Family Likert Scale

***Pedals Possible Family Questionnaire***

**For the following phrases, please circle the response that you most identify with:**

**Overall Impression of Program**

1. My child had a positive experience with the school-based adaptive cycling program.

Strongly Agree Agree Neutral Disagree Strongly Disagree

1. I would sign my child up for a program like this again.

Strongly Agree Agree Neutral Disagree Strongly Disagree

1. I would recommend this program to other school districts.

Strongly Agree Agree Neutral Disagree Strongly Disagree

**Family/Social Engagement**

1. My child shows improved engagement with family in home and in daily tasks (meal time, chores, self-care, etc).

Strongly Agree Agree Neutral Disagree Strongly Disagree

1. My child shows improved participation in social settings with peers and in the community.

Strongly Agree Agree Neutral Disagree Strongly Disagree

**Physical Activity**

1. My child’s ability to perform gross motor tasks has improved (walking, running, wheeling wheelchair, riding bike, etc.).

Strongly Agree Agree Neutral Disagree Strongly Disagree

1. My child has improved participation in physical activity during the day (sports, playing on playground, outside play, etc.).

Strongly Agree Agree Neutral Disagree Strongly Disagree

1. My child has an improved mood.

Strongly Agree Agree Neutral Disagree Strongly Disagree

Overall Comments:­_______________________________________________________________________________________________________________________________________­­­

## Pre- and Post- Intervention Measures Flowsheet and GAS examples

**LCSD “Pedals Possible” Pre and Post-Assessment Flowsheet**

| Date |  |
| --- | --- |
| Student # |  |
| Student Age (year/month) |  |
| School |  |
| Teacher/Therapist Last Name |  |

**Goal Attainment Scale (GAS) Template & 3 Example goals: (highlighted at post)**

| **Attainment Level** | **Description** |
| --- | --- |
| Baseline | Student cycles for 30 seconds prior to needing a rest break. |
| Less than expected outcome | Student cycles for 60 seconds prior to needing a rest break. |
| Expected outcome | Student cycles for 90 seconds prior to needing a rest break. |
| Greater than expected outcome | Student cycles for 120 seconds prior to needing a rest break. |
| Much greater than expected outcome | Student cycles for 150 seconds prior to needing a rest break. |

| **Attainment Level** | **Description** |
| --- | --- |
| Baseline | When riding on the bike for 6 minutes, student requires 5 verbal/tactile cues to keep hands on the handlebars. |
| Less than expected outcome | When riding on the bike for 6 minutes, student requires 4 verbal/tactile cues to keep hands on the handlebars. |
| Expected outcome | When riding on the bike for 6 minutes, student requires 3 verbal/tactile cues to keep hands on the handlebars. |
| Greater than expected outcome | When riding on the bike for 6 minutes, student requires 2 verbal/tactile cues to keep hands on the handlebars. |
| Much greater than expected outcome | When riding on the bike for 6 minutes, student requires 1 verbal/tactile cue to keep hands on the handlebars. |

| **Attainment Level** | **Description** |
| --- | --- |
| Baseline | During the 3-hour school day, student will successfully pick/indicate the “bike” card when presented with 2 cards on 0/5 trials. |
| Less than expected outcome | During the 3-hour school day, student will successfully pick/indicate the “bike” card when presented with 2 cards on 1/5 trials. |
| Expected outcome | During the 3-hour school day, student will successfully pick/indicate the “bike” card when presented with 2 cards on 2/5 trials. |
| Greater than expected outcome | During the 3-hour school day, student will successfully pick/indicate the “bike” card when presented with 2 cards on 3/5 trials. |
| Much greater than expected outcome | During the 3-hour school day, student will successfully pick/indicate the “bike” card when presented with 2 cards on 4/5 trials. |

**6-Minute Adaptive Cycling Test Distance**  ___________feet

Assistance Level Required

1. Steering Assist?

No Assistance Min (25% help) Mod (50% help) Max(75% or more help)

1. Pedal Assist?

No Assistance Min (25% help) Mod (50% help) Max(75% or more help)

1. What other physical supports were used (ex: chest strap, other supports)? ____________________

## 1.4 Daily Flowsheet (front page)

**LCSD “Pedals Possible” Adaptive Cycling Treatment Flowsheet**

| Date |  |
| --- | --- |
| Student # |  |
| Student Age (year/months) |  |
| Teacher/Therapist Name |  |
| School |  |
| Session Start Time |  |
| Session End Time |  |

**Remember to put on the student’s Seat Belt and Helmet!!**

**Pre-Treatment Measures**

**Face, Legs, Activity Cry, Consolability (FLACC) Behavioral Pain Scale** (circle one number in each category)

| **Categories** | **0** | **1** | **2** |
| --- | --- | --- | --- |
| **Face** | No particular expression OR smile | Occasional grimace or frown; withdrawn, disinterested | Frequent to constant frown, clenched jaw, quivering chin |
| **Legs** | Normal position OR relaxed | Uneasy, restless, tense | Kicking OR legs drawn up |
| **Activity** | Lying quietly, normal position, moves easily | Squirming, shifting back and forth, tense | Arched, rigid, or jerking |
| **Cry** | No cry | Moans or whimpers, occasional complaint | Crying steadily, screams or sobs; frequent complaints |
| **Consolability** | Content, relaxed | Reassured by occasional touching, hugging, or being talked to; distractible | Difficult to console or comfort |
| **TOTAL Score** |  |  |  |

**IF TOTAL SCORE IS GREATER THAN 8, NO CYCLING IS TO BE COMPLETED WITH THE STUDENT**

**Happiness Scale** (Please circle one based on observation or interview with student)


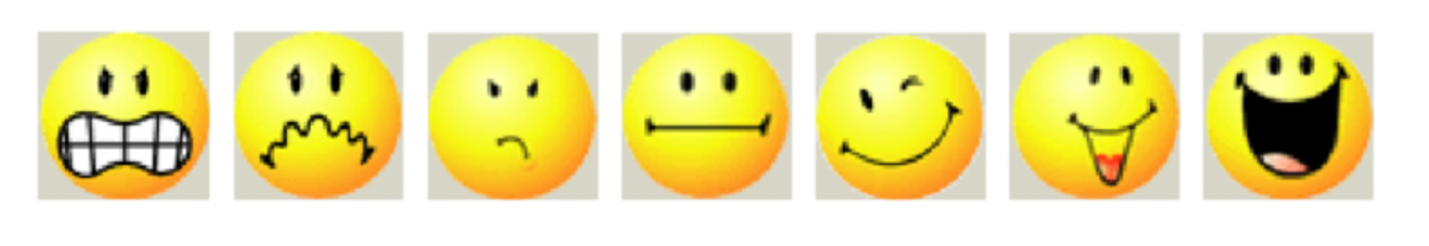


**Post-Treatment Measures**

| **Cycle Computer Data** | **Measurement** |
| --- | --- |
| Cycling Duration (Time spent actually cycling) | Minutes: Seconds |

**Assistance Level Required**

1. Steering Assist?

No Assistance Min (25% help) Mod (50% help) Max(75% or more help)

1. Pedal Assist?

No Assistance Min (25% help) Mod (50% help) Max(75% or more help)

1. What other physical supports were used (ex: chest strap, other supports)? ________________________________________________________________________

**Face, Legs, Activity Cry, Consolability (FLACC) Behavioral Pain Scale** (circle one number in each category)

| **Categories** | **0** | **1** | **2** |
| --- | --- | --- | --- |
| **Face** | No particular expression OR smile | Occasional grimace or frown; withdrawn, disinterested | Frequent to constant frown, clenched jaw, quivering chin |
| **Legs** | Normal position OR relaxed | Uneasy, restless, tense | Kicking OR legs drawn up |
| **Activity** | Lying quietly, normal position, moves easily | Squirming, shifting back and forth, tense | Arched, rigid, or jerking |
| **Cry** | No cry | Moans or whimpers, occasional complaint | Crying steadily, screams or sobs; frequent complaints |
| **Consolability** | Content, relaxed | Reassured by occasional touching, hugging, or being talked to; distractilble | Difficult to console or comfort |
| **TOTAL Score** |  |  |  |

**Happiness Scale** (Please Circle One)

**
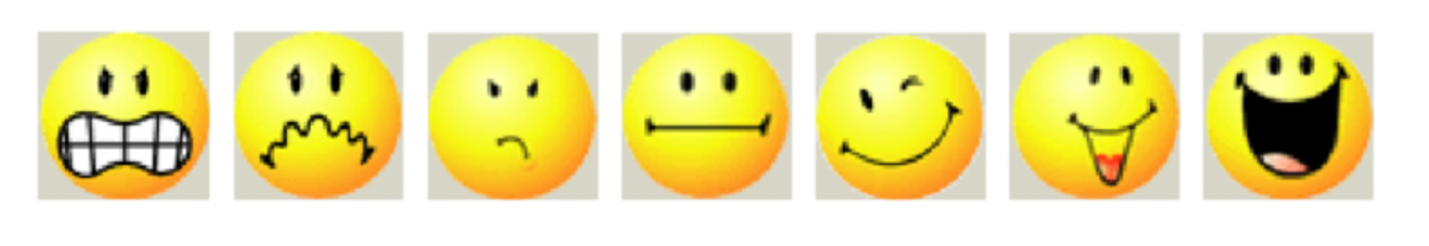
**

Additional Subjective Comments: _____________________________________________________________________________________

_____________________________________________________________________________________

# Supplementary Figures and Tables

**2.1 Summary of participant recruitment**
